# Editorial: Contemporary Perspective on 5-HT_2C_ Receptor Function and Its Pharmacological Targeting

**DOI:** 10.3389/fphar.2020.606414

**Published:** 2020-11-23

**Authors:** Philippe De Deurwaerdère, Abdeslam Chagraoui, Kathryn A. Cunningham

**Affiliations:** ^1^ Institut des Neurosciences Cognitives et Integratives d’Aquitaine, CNRS UMR 5287, Bordeaux, France; ^2^ Neuronal and Neuroendocrine Differentiation and Communication Laboratory, Institute for Research and Innovation in Biomedicine of Normandy (IRIB), Normandie University, UNIROUEN, INSERM, U1239, Rouen, France; ^3^ CHU Rouen, Rouen, France; ^4^ Center for Addiction Research and Department of Pharmacology and Toxicology, University of Texas Medical Branch, Galveston, TX, United States

**Keywords:** addiction, monoamines, polymorphism, mood disorder, mRNA editing, peptidomimetic, cell signaling, post-traumatic syndrome disorder

This compilation of research and review articles presents contemporary perspectives dedicated to serotonin (5-HT) 5-HT_2C_ receptor (5-HT_2C_R) and its pharmacological targeting. The volume also covers the impact of disease conditions or treatments on 5-HT_2C_R expression and function in preclinical models of human pathologies. The 5-HT_2C_R is indeed an intriguing and fascinating G protein-coupled receptor (GPCR) with amazing regulatory features devised by biology, including its pre-transcriptional regulation, alternative splice variants and post-transcriptional RNA editing. RNA editing is an important molecular process from a pharmacological perspective because of the generation of multiple 5-HT_2C_R isoforms with distinct pharmacological properties. Most recently, the 5-HT_2C_R has been shown to exist in oligomeric formations and the 5-HT_2C_R is thought to signal as homodimer and could function as a heterodimer in complex with other GPCRs with the consequence of changing the molecular and cellular impact of the receptor signaling. The allure of uncovering the scientific secrets of the 5-HT_2C_R system lies in the prospects 5-HT_2C_R ligands as potential therapeutics given its value as a pharmacological target in a number of neuropsychiatric diseases including eating disorders, drug addiction, schizophrenia, mood disorders, impulse control disorders, obsessive compulsive disorder, and epilepsy (Howell and Cunningham, 2015; Chagraoui et al., 2016; Di Giovanni and De Deurwaerdère, 2016).

Some medications employed in the clinic bind to the 5-HT_2C_R including some atypical antipsychotics, the antidepressants agomelatine, mianserin and mirtazapine, and lorcaserin which achieved the most advanced drug targeting 5-HT_2C_R to reach the clinic. However, lorcaserin was voluntarily removed from the market in 2020 due to an FDA safety communication. At this time, there are no FDA-approved 5-HT_2C_R-selective agonists, however, lorcaserin was marketed to promote weight loss in patients with a body mass index of greater than 30 or with a BMI of greater than 27 comorbid with type-2 diabetes, hypertension, or dyslipidemia. Lorcaserin is emblematic for at least four reasons in neuropharmacology and in the field of 5-HT_2C_R research. First, it achieved status as an anti-obesity product issued from years of intensive research aimed at deciphering the mechanism of action underlying the anorexigenic effect of d-fenfluramine, thereby identifying the 5-HT_2C_R as one of the main actors. Second, upon its accession to the clinic, lorcaserin became emblematic of clinical and preclinical orientations to repurpose drugs away from their initial purpose; in the case of lorcaserin, preclinical studies predict the efficacy of a 5-HT_2C_R-selective agonist for treatment of drug addiction and impulse control disorders (Higgins et al., 2020). Some of the preclinical arguments are presented herein in the article by Soto et al. who report that lorcaserin or the investigatory 5-HT_2C_R agonist WAY-163,909 reduces the ability of trained rats to discriminate cocaine from saline (Soto et al., 2019). Third, even though a drug like lorcaserin could be selected to fulfill a hypothesized mechanism of action in the treatment of drug abuse, e.g., to oppose dopaminergic transmission in the reward system, its mechanism of action has some shadowy aspects. For instance, Di Giovanni et al. recall that lorcaserin has mitigated effects on dopamine extracellular levels. However, they report that lorcaserin changes the profile and the number of correlations of postmortem monoamines tissue markers across 30 brain areas toward a decrease for dopamine, and an increase for serotonin and noradrenaline (Di Giovanni et al., 2020). Thus beyond the dopaminergic system, 5-HT_2C_R agonists modulate the other monoaminergic systems which could permit to develop multi-drug design to boost the efficacy of 5-HT_2C_R agonists. Fourth, at a time where lorcaserin was under study in clinical protocols for diseases other than those afferent to obesity, lorcaserin was withdrawn from the market due to suspected cases of cancer. Thus, lorcaserin studies have confirmed that 5-HT_2C_R targeting was valuable in neuropsychiatric diseases where there is no clear treatment, opening the path to develop new pharmacological strategies. The real purpose of the article of Soto et al. is to emphasize one of these strategies from the peptide design to the behavioral studies in the context of cocaine addiction. Briefly, they show that disruption of the 5-HT_2C_R interaction with the protein phosphatase and tensin homolog (PTEN) via peptidomimetics enhances 5-HT_2C_R-mediating signaling *in vitro* and potentiates selective 5-HT_2C_R agonists in behavioral rodent models.

The search continues particularly in the field of individual responses to drug of abuse. In their review article, Tanaka and Watanabe elaborate on the editing of the 5-HT_2C_R mRNA and alcohol consumption. They recall the molecular origins of the 5-HT_2C_R mRNA editing with the involvement of the deaminated to inosine by adenosine deaminase enzymes comprising three subtypes isolated so far. They summarize the data showing that the distribution of the various 5-HT_2C_R edited products varies across brain regions, mammal species and strain, and pathophysiological conditions. The authors focus herein on alcohol preference in different mouse strains and a possible abnormal editing of the 5-HT_2C_R mRNA in the nucleus accumbens. That the activity of 5-HT_2C_R mRNA editing could impact behavioral traits and accompany pathophysiological states is highlighted by the transgenic mouse model used by Paizanis et al. in which only the full edited 5-HT_2C_R product VGV is expressed (Paizanis et al., 2020). These transgenic mice exhibit anxiety and abnormal fear conditioning and contextualized learning, modeling some features of the post-traumatic stress disorder. In contrast to the expectations of the authors, VGV mice do not exhibit enhanced voluntary alcohol and cocaine consumption. Rather, alcohol and cocaine exhibited some potential therapeutic effects in VGV mice, normalizing the expression of brain-derived neurotrophic factor mRNA expression in the hippocampus compared to wildtype mice.

The link between 5-HT_2C_R mechanisms and anxiety and mood disorders has been acknowledged for several years but requires additional neurobiological exploration to align the proper pharmacological modification of 5-HT_2C_R required for therapeutic improvement (Chagraoui et al., 2016; Di Giovanni and De Deurwaerdère, 2016). This complexity is possibly related to the distinct sites of 5-HT_2C_R expression in the brain which participate in the regulation of mood and cognition. Baptista-de-Souza et al. focused on 5-HT transmission within the amygdala and the periaqueductal gray matter (PAG) of mice in the context of fear-induced anti-nociception. In these studies, treatment with the selective serotonin reuptake inhibitor (SSRI) fluoxetine impaired the ability of the 5-HT_2C_R or 5-HT_1A_R within the amygdala and PAG to intensify nociceptive responses to aversive stimuli. Papp et al. studied the alterations of the gamma power of the electroencephalogram (EEG) in rats as a suggested biomarker of depression. Correspondingly, quantitative EEG analysis revealed that acute treatment with the selective 5-HT_2C_R antagonist SB-242,084 mimicked chronic treatment with the SSRI escitalopram in increasing gamma power (30–60 Hz) in light and deep slow-wave sleep. This profile prompted the authors to conclude that SB-242,084 may potentially be a useful antidepressant.

Some single nucleotide polymorphisms (SNPs) in the X-linked *HTR2C* gene have been correlated with aggressive behavior, antipsychotic drug-induced hyperprolactinemia and tardive dyskinesia as recalled by (Ochi et al., 2019). It is believed that these SNPs could either accompany pathological states and/or the efficacy of treatments. However, Ochi et al. report that, in a human cohort treated for major depressive disorder, no associations between the efficacy of antidepressant drugs (SSRI or tricyclic antidepressants) and several 5-HT receptor genes, including those concerning the *HT2CR* SNPs. The authors do not exclude the possibility that differential responses to antidepressants in males vs. females may relate to specific 5-HT receptor SNPs as the sample size was limited, impairing sub-analysis of the data.

In conclusion of this collection of articles, 5-HT_2C_R remains an attractive target for the treatment of several chronic health disorders. Future studies that interrogate this unique receptor from its transcription to integrative neurobiology are required to target therapeutics for normalization of 5-HT_2C_R function in disease states. Importantly, the classical means of conceiving treatments with agonists, antagonists, and biased agonists has already been implemented in 5-HT_2C_R research by allosteric modulators (Wold et al., 2019) acting on specific site of the receptor (Wold et al., 2020). It could be further implemented by treatments specifically targeting transcription, translation, and intracellular signaling. The clinical data are important to elaborate on the participation of 5-HT_2C_R in a given pathology and/or in response to treatments. For instance, the 5-HT_2C_R-selective agonist vabicaserin was shown to suppress activated central dopaminergic transmission, which served as the basis for the clinical trial in schizophrenia (Shen et al., 2014). The reported findings supported the safety, tolerability, and efficacy of vabicaserin in acute schizophrenia, but future studies are required to firmly establish the role of central dopaminergic systems in this outcome in humans. Another dimension which is not treated in this collection is that a multi-target approach combining 5-HT_2C_R ligands and compounds targeting other sites (for instance the combination 5-HT_2C_R agonist and 5-HT_2A_R antagonist in cocaine addiction (Howell and Cunningham, 2015) could offer broader and safer spectrums of clinical efficacy. Thus, additional studies are needed for establishing 5-HT_2C_R biological roles alone or in interaction with other systems in the regulation of the activity of neurobiological networks of the whole central nervous system. Clearly, there are rich opportunities for several other collections on this intriguing topic in the future.

## Author Contributions

All authors listed have made a substantial, direct, and intellectual contribution to the work and approved it for publication.

## Conflict of Interest

The authors declare that the research was conducted in the absence of any commercial or financial relationships that could be construed as a potential conflict of interest.
